# Sebaceous carcinoma of the skin of the breast: a case report

**DOI:** 10.1186/1752-1947-2-276

**Published:** 2008-08-15

**Authors:** Ahmed Alzaraa, Imran Ghafoor, Andrew Yates, Alhad Dhebri

**Affiliations:** 1 Department of General Surgery, Tameside General Hospital, Manchester, UK; 2 Department of Histopathology, Tameside General Hospital, Manchester, UK

## Abstract

**Introduction:**

Sebaceous gland tumours are rare and their presence should be considered as a marker for Muir-Torre Syndrome, alerting to search for an occult malignancy.

**Case presentation:**

A 43-year-old Caucasian female patient underwent excision of a sebaceous cyst. Histopathology confirmed a sebaceous carcinoma. Further investigations revealed multiple intra-abdominal malignancies. She has been under regular follow-up in the relevant clinics.

**Conclusion:**

Sebaceous carcinoma should be excised completely and followed-up for the detection of possible metastases. Surgical removal of primary or metastatic cancers may be curative and should be attempted wherever possible. It is very important for clinicians not to miss such skin lesions as they may precede the presentation of internal malignancies.

## Introduction

Muir-Torre Syndrome (MTS) is defined by the combination of a sebaceous gland tumour and at least one visceral carcinoma occurring in the same individual in the absence of other precipitating factors such as radiotherapy or AIDS. Typical skin lesions associated with this syndrome include sebaceous adenoma, epithelioma and carcinoma.

## Case presentation

A 43-year-old Caucasian woman had an excision of an infected sebaceous cyst from the skin of her left breast in 2007. Histopathology reported an incompletely excised sebaceous carcinoma, suggestive of MTS. The patient was referred to the breast clinic for further assessment. She had an extensive family history of cancers. Her father died of prostate cancer, her mother had a hysterectomy for uterine cancer, her maternal grandmother had uterine cancer, her sister had bowel cancer and her great paternal aunt had breast cancer. Clinical examination revealed a scar in the lower half of her left breast and two sebaceous cysts over her left nipple and left upper arm. She was also tender in the right iliac fossa and was investigated by a gynaecologist. Her chest X-ray and bilateral mammogram were normal. The patient underwent wider excision of the left breast scar and of skin lesions over the left nipple and the left upper arm. Histology reported no residual neoplasia in the excised scar and the skin lesions were benign epidermoid cysts.

A computed tomography (CT) scan of the chest, abdomen and pelvis showed bilateral ovarian cysts and diverticular disease of the colon. The tumour markers were raised: CA125: 149 (0–35); CA19-9: 61.8 (0–35). She later underwent total abdominal hysterectomy, bilateral salpingo-oophorectomy and infracolic partial omentectomy. The histology was Stage IC moderately differentiated endometrioid adenocarcinoma of the right ovary, Stage IC endometrioid endometrial adenocarcinoma, and the omentum was normal. She was referred to a geneticist, oncologist, gastroenterologist and dermatologist. The genetic analysis showed (MSH2s.1578DEL). She had a gastroscopy which showed duodenitis and a colonoscopy which was normal. She had chemotherapy followed by radiotherapy.

## Discussion

Cutaneous lesions associated with hereditary cancer syndromes are known as cancer-associated genodermatoses [1]. One of these is MTS, defined by the combination of a sebaceous gland tumour and at least one visceral carcinoma occurring in the same individual in the absence of other precipitating factors such as radiotherapy or AIDS [2]. It was first described by Muir in 1967 and Torre in 1968, and is recognized as a subtype of Lynch Type II Hereditary Nonpolyposis Colon Cancer (HNPCC) [3,4]. A review by Akhtar *et al*. (1999) identified a total of 205 reported cases in the world literature [5]. The male/female ratio is 3:2 [6]. It is an autosomal dominant disorder with a high degree of penetrance and variable expression and the children of an affected individual have a 50% risk of inheriting the cancer predisposition. The genetic disorder is an inherited germline mutation in one of the DNA mismatch repair (MMR) genes, most commonly MSH2, which eventually leads to microsatellite instability (MSI) [7].

Typical skin lesions associated with this syndrome include sebaceous adenoma, epithelioma and carcinoma (sebaceous hyperplasia and nevus sebaceous of Jadassohn are generally excluded). Keratoacanthomas and basal cell carcinomas with sebaceous differentiation can also occur. Sebaceous gland tumours are rare and their presence should be considered as a marker for MTS, alerting to search for an occult malignancy [8]. Skin lesions may precede the presentation of internal malignancies, but often develop later. Fifty-six percent of skin lesions occur after the diagnosis of the first malignancy, 6% occur concomitantly and 22% occur as the first malignancy of the syndrome [5]. The cutaneous lesion may occur as much as 25 years before or 37 years after the internal malignancy. Multiple primary carcinomas at different sites are characteristic of MTS (Table 1), and up to nine visceral cancers have been reported in one individual [9].

**Table 1:** Prevalence of cancers associated with MTS

Cancer	Percentage (%)
Colorectal	80
Stomach	11–19
Hepatobiliary tract	2–7
Small intestine	1–4
Brain or central nervous system	1–3
Endometrial	20–60
Ovarian	9–12
Urinary tract	4–5
Skin	Increased risk

Colorectal cancer is the commonest visceral neoplasm to occur in MTS, and the most frequent initial cancer [10,11], though not in our case. In common with other forms of HNPCC, colorectal cancers in MTS are usually proximal in location and tend to have a more indolent course than usual colorectal cancers [11]. Fifty-one percent of MTS patients develop at least one colorectal cancer, and multiple colorectal cancers are common [12]. Colonic polyps are found in more than 25% of MTS patients, and are especially prevalent in patients with colorectal carcinoma [12].

The second most common site is the genitourinary tract (including endometrium and ovary), representing approximately one-quarter of visceral cancers. A wide variety of other cancers have been reported involving breast, upper gastrointestinal tract, upper respiratory tract including larynx, salivary gland, and haematological malignancies including lymphoma and leukaemia. Intestinal polyps occur in at least one-quarter of patients [12].

In families with proven germline mutation, individuals should be offered regular screening examinations. In those who can be demonstrated not to have inherited the germline mutation, cancer surveillance is not necessary [13]. Screening for malignancy at all possible sites is impractical in MTS given the wide range of associated malignancies, and should probably concentrate on the colorectum, female genital tract and possibly the renal tract. In some families, the occurrence of certain other tumours would be an indication for other screening modalities, for example, upper gastrointestinal endoscopy [14]. Cohen et al. [11] suggested that a search for internal malignancies should be undertaken in those with MTS-associated sebaceous gland tumour, those with MTS, and in family members of an MTS patient. They also suggested a surveillance programme for patients with MTS or MTS-associated sebaceous gland tumours including annual clinical examination, carcinoembryonic antigen (CEA), cervical smear, chest radiography, urine cytology, colonoscopy or barium enema every 3 to 5 years, and for female patients, mammography annually or biennially to age 50 and annually thereafter, and endometrial biopsy every 3 to 5 years. Other authors have suggested that colonoscopy should be more frequent in view of the high frequency of colonic cancer and its proximal predominance, and have advocated annual colonoscopy from the age of 25 years [10]. MTS screening may be extensive and is not always performed. A search for mutations in either MSH1 or MSH2 is expensive and time consuming. One could analyse MMR protein expression as a surrogate for assaying for the respective gene mutations [15]. Benign sebaceous tumours and keratoacanthomas can be conservatively treated with excision or cryotherapy. Sebaceous carcinoma should be excised completely and followed-up for the detection of possible metastases. It has been suggested that, because of their relatively good prognosis and non-aggressive course, surgical removal of primary or metastatic cancers may be curative and should be attempted wherever possible [5,7]. The combination of interferon with retinoids (isotretinoin) seems to be of promise in preventing tumour development in MTS. A dosage of 0.8 mg/kg/day may be effective [10].

## Conclusion

Sebaceous gland tumours are rare and their diagnoses should suggest the possibility of MTS and prompt a search for associated malignancies, and for the underlying genetic mutation. Family members should be offered screening to detect early cancers. Genetic studies can help identify the inherited molecular defect that causes MTS.

## Abbreviations

AIDS: Acquired Immune Deficiency Syndrome; CA125: Cancer Antigen 125; CA19-9: Cancer Antigen 19-9; CEA: carcinoembryonic antigen; CT: computed tomography; DNA: deoxyribonucleic acid; HNPCC: Hereditary Non-Polyposis Colon Cancer; MMR: mismatch repair; MSI: microsatellite instability; MTS: Muir-Torre Syndrome.

## Competing interests

The authors declare that they have no competing interests.

## Authors' contributions

AA searched the literature and drafted the manuscript, IG searched the literature, AY evaluated the histology, and AD operated on the patient and edited the manuscript.

**Figure 1:**
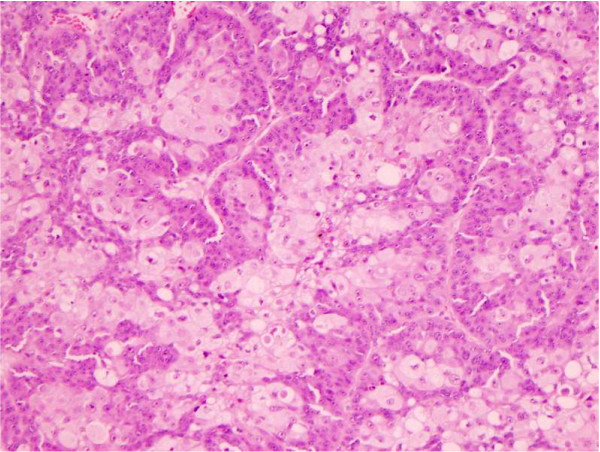
Sebaceous carcinoma showing multivacuolated cells with clear cytoplasm and indented nuclei as evidence of sebaceous differentiation.
